# Supplementary material for: Impact of the scale-up of piped water on urogenital schistosomiasis infection in rural South Africa
Source: eLife. 2018 Feb 20;7:e33065. doi: 10.7554/eLife.33065 (PMC5819946; doi:10.7554/eLife.33065)
Supplement: Supplementary file 1. — Model 0 gives the univariate results and Model 1 includes all variables in the model. In Model 2, piped water coverage in the immediate community surrounding each participant has been substituted with the household-level piped water covariate. [file elife-33065-supp1.docx]

† Computes the proportion of households having access to piped-water in the unique community surrounding each participant in the study (**Figure 3**). The Quintile (Q) ranges (min–max) are: Q1: 0–36; Q2: 37–59; Q3: 60–75; Q4: 76–92; Q5: 93–100 ‡ Corresponding values for a model in which community-level piped-water coverage is used as a continuous variable: a 1% increase in the coverage of piped-water in the surrounding community, was independently associated with a 1.7% decrease in the risk of a *Schistosoma haematobium* infection (aPR=0.983; 95% CI: 0.977, 0.989; p-value<0.001).

†Derived using a standard Gaussian kernel (radius 2 km) applied to households in the immediate community surrounding each participant in the study (**Figure 2f**).
