# Supplementary material for: Impact of the scale-up of piped water on urogenital schistosomiasis infection in rural South Africa
Source: eLife. 2018 Feb 20;7:e33065. doi: 10.7554/eLife.33065 (PMC5819946; doi:10.7554/eLife.33065)
Supplement: Supplementary file 2. — Model 0 gives the univariate results and Model 1 gives the multivariate results for the availability of piped water in the community. Model 2 shows the instrumental variable estimation (IVE) results corresponding to Model 1, where the instrumental variable is the year that piped water was introduced into the community. Model 3 gives the multivariate results for piped water in the household and Model 4 shows the corresponding IVE results. [file elife-33065-supp2.docx]

† Computes the proportion of households having access to piped water using a standard Gaussian kernel applied to households in the unique community surrounding each participant in the study (**Figure 3**).

†Derived using a standard Gaussian kernel (radius 2 km) applied to households in the immediate community surrounding each participant in the study (**Figure 2f**).
