# Supplementary material for: Impact of the scale-up of piped water on urogenital schistosomiasis infection in rural South Africa
Source: eLife. 2018 Feb 20;7:e33065. doi: 10.7554/eLife.33065 (PMC5819946; doi:10.7554/eLife.33065)
Supplement: Transparent reporting form [file elife-33065-transrepform.docx]

***eLife’s* transparent reporting form**

We encourage authors to provide detailed information *within their submission* to facilitate the interpretation and replication of experiments. Authors can upload supporting documentation to indicate the use of appropriate reporting guidelines for health-related research (see [EQUATOR Network](http://www.equator-network.org/%20)), life science research (see the [BioSharing Information Resource](https://biosharing.org/)), or the [ARRIVE guidelines](http://www.plosbiology.org/article/info:doi/10.1371/journal.pbio.1000412) for reporting work involving animal research. Where applicable, authors should refer to any relevant reporting standards documents in this form.

If you have any questions, please consult our Journal Policies and/or contact us: [editorial@elifesciences.org](mailto:editorial@elifesciences.org).

**Sample-size estimation**

- You should state whether an appropriate sample size was computed when the study was being designed
- You should state the statistical method of sample size computation and any required assumptions
- If no explicit power analysis was used, you should describe how you decided what sample (replicate) size (number) to use

Please outline where this information can be found within the submission (e.g., sections or figure legends), or explain why this information doesn’t apply to your submission:

The assumptions behind the sample size calculation, can be found in the “sample size” section of the methods on pages 17 and 18 of the manuscript.

The sample size calculation was based on the number of participants required to test the impact of access to piped-water on infection with *Schistosoma haematobium* stratified by sex; as well as provide good geographical coverage of the surveillance area. Given two grades sampled at each of the 33 schools (50 pupils per grade) in the study area and an assumption of 70% consent and 90% linkage to the population cohort, we would attain an estimated sample size of 2,079. We then assumed that the piped-water exposure occurred in 50% of participants and that 30% of pupils tested for *Schistosoma haematobium* were infected. This prevalence estimate was based on historical parasitological surveys in surrounding areas, more recent surveys in the province of KwaZulu-Natal and expected reductions in prevalence as a result of the piped water roll-out in the study area during the preceding decade. Under these assumptions, we would attain 99% power to detect a relative risk of ≥1.5 (95% CI) in boys and girls respectively as well as provide good geographical coverage of the study area to facilitate identification of micro-geographical clustering of infections.

**Replicates**

- You should report how often each experiment was performed
- You should include a definition of biological versus technical replication
- The data obtained should be provided and sufficient information should be provided to indicate the number of independent biological and/or technical replicates
- If you encountered any outliers, you should describe how these were handled
- Criteria for exclusion/inclusion of data should be clearly stated
- High-throughput sequence data should be uploaded before submission, with a private link for reviewers provided (these are available from both GEO and ArrayExpress)

Please outline where this information can be found within the submission (e.g., sections or figure legends), or explain why this information doesn’t apply to your submission:

This information is not required for this submission because our study consisted of a single parasitological survey and the analysis thereof (described in detail in the manuscript).

**Statistical reporting**

- Statistical analysis methods should be described and justified
- Raw data should be presented in figures whenever informative to do so (typically when N per group is less than 10)
- For each experiment, you should identify the statistical tests used, exact values of N, definitions of center, methods of multiple test correction, and dispersion and precision measures (e.g., mean, median, SD, SEM, confidence intervals; and, for the major substantive results, a measure of effect size (e.g., Pearson's r, Cohen's d)
- Report exact p-values wherever possible alongside the summary statistics and 95% confidence intervals. These should be reported for all key questions and not only when the p-value is less than 0.05.

Please outline where this information can be found within the submission (e.g., sections or figure legends), or explain why this information doesn’t apply to your submission:

A description of all statistical tests carried out in this research can be found in the statistical analysis section on page 21 and 22 of the manuscript. All effect sizes, exact p-values, confidence intervals and numbers per sub-group are provided in tables 1-3, S1, S2 of the manuscript.

(For large datasets, or papers with a very large number of statistical tests, you may upload a single table file with tests, Ns, etc., with reference to sections in the manuscript.)

**Group allocation**

- Indicate how samples were allocated into experimental groups (in the case of clinical studies, please specify allocation to treatment method); if randomization was used, please also state if restricted randomization was applied
- Indicate if masking was used during group allocation, data collection and/or data analysis

Please outline where this information can be found within the submission (e.g., sections or figure legends), or explain why this information doesn’t apply to your submission:

Not applicable

**Additional data files (“source data”)**

- We encourage you to upload relevant additional data files, such as numerical data that are represented as a graph in a figure, or as a summary table
- Where provided, these should be in the most useful format, and they can be uploaded as “Source data” files linked to a main figure or table
- Include model definition files including the full list of parameters used
- Include code used for data analysis (e.g., R, MatLab)
- Avoid stating that data files are “available upon request”

Please indicate the figures or tables for which source data files have been provided:

Nil
